# Prognostic analysis of early-stage squamous cell carcinoma of the vulva

**DOI:** 10.1186/1477-7819-11-20

**Published:** 2013-01-26

**Authors:** Li-Qun Xu, Rong-Zhen Luo, Xue-Ming Sun, Jie-Hua He, Yan-Na Zhang

**Affiliations:** 1 Department of Gynecology, State Key Laboratory of Oncology in South China, Sun Yat-sen University Cancer Center, Guangzhou, Guangdong, 510060, P. R. China; 2 Department of Pathology, State Key Laboratory of Oncology in South China, Sun Yat-sen University Cancer Center, Guangzhou, Guangdong, 510060, P. R. China; 3 Department of radiotherapy, State Key Laboratory of Oncology in South China, Sun Yat-sen University Cancer Center, Guangzhou, Guangdong, 510060, P. R. China

**Keywords:** Vulvar squamous cell carcinoma, Surgical, Prognosis

## Abstract

**Aim:**

The aim of this study was to analyze prognostic factors of early-stage squamous cell carcinoma of the vulva.

**Methods:**

A retrospective analysis was conducted on 35 patients who were treated for early-stage squamous cell carcinoma of the vulva at Sun Yat-sen University Cancer Center from January 1980 to December 2005. The Statistical Package for Social Science (SPSS) was used to compare the different strategies of operation and to analyze the prognostic factors.

**Results:**

Thirty-five patients had early-stage squamous cell carcinoma of the vulva. Of these cases, 26 were well differentiated, seven were moderately differentiated, and two were poorly differentiated. The five-year survival rate was 77.1%. Five cases were in FIGO stage 1a and 30 cases were in stage 1b; median survival times were 182.3 months and 152.5 months, and the five-year survival rates were 100% and 81.5% (*P* >0.05), respectively. The five-year survival of the patients who underwent local excision; radical vulvectomy and en bloc resection of inguinofemoral lymphadenectomy; orradical vulvectomyen bloc resection of inguinofemoral lymphadenectomy, and pelvic lymph nodes was 50%, 81.8%, and 83.9%, respectively. For these cases, 74.3% of the tumors were medial while 25.7% were lateral, and the five-year survival rates of patients according to tumor location were 87.0% and 64.8% (*P* <0.05), respectively. The inguinal lymph node not increased and active were 16 cases (45.7%), and increased, active and hard were 17 cases (48.6%), and syncretic were two cases (5.7%), five-year survival rates were 73.3%, 92.9% and 50% (P <0.05), respectively. Of these cases, 74.3% of the tumors were cauliflower-like and 25.7% were nodular; five-year survival rates by tumor type were 91.3% and 66.7% (*P* <0.05), respectively.

**Conclusions:**

For patients with early-stage squamous cell carcinoma of the vulva, surgical operation is the primary, yet the best, treatment. The related prognostic factors were tumor location (lateral/medial), stage, gross morphology, and clinical state of the inguinal lymph node.

## Background

In primary gynecological malignancies, vulvar carcinoma is relatively rare, constituting approximately 3% to 5%. One of the most common types of vulvar carcinoma is squamous cell carcinoma, which accounts for about 90% of malignant tumors, whereas melanoma, basal cell carcinoma, Pap adenocarcinoma, sarcoma, and Paget’s disease are much less common [1]. Early vulvar squamous cell carcinoma has not been taken seriously enough for lack of obvious symptoms.

Of the 150 patients with confirmed vulvar squamous cell carcinoma who were treated in our cancer center, 35 cases (23%) were early carcinoma (stage I). In recent years, many scholars have advocated for implementing individualized surgical treatment with different operations according to the position, depth of invasion and other risk factors in early vulvar squamous cell carcinoma cases. But identification of early incidence is relatively low, fewer cases are reported, and operation mode and prognostic factors still incite controversy. This paper sums up the clinical data of early vulvar squamous cell carcinoma for 20 years with the objective of evaluating the impact of clinicopathologic prognostic factors and different surgical methods on early-stage vulvar squamous cell carcinoma prognosis.

## Methods

From January 1980 to December 2005, 35 patients with early-stage invasive squamous cell carcinoma of the vulva presented to Sun Yat-sen University Cancer Center, of which three cases were complicated with hypertension and diabetes mellitus. In these 35 cases, patient ages ranged from 30 to 79 years old (median 59.2 years). Upon presentation, patients who complained of vulva pruritus or neoplasm accounted for 91.4%, their symptoms appeared a median time of 72.2 months earlier (ranging from one month to 20 years), and the follow-up time was 3.7 to 297.7 months (median 79.7). Vulvar biopsy confirmed white spot lesions for 60% of the cases.

For these cases 74.3% of the tumors were medial type while 25.7% were lateral type. Determination of disease stage was performed according to the International Federation of Gynecology and Obstetrics (FIGO) Criteria 2009, of which five cases were in stage 1a and 30 cases were in stage 1b.

Only one case, with a large vulvar tumor (maximum diameter of 4 cm) near the urethra, received one cycle of chemotherapy preoperatively and two cycles postoperatively; the remaining cases underwent surgical treatment alone. The number of patients who underwent local excision; radical vulvectomy and en bloc resection of inguinofemoral; or radical vulvectomy, en bloc resection of inguinofemoral, and pelvic lymph nodes was two, 12 and 21 cases, respectively.

Overall survival was registered according to the time interval from diagnosis to death due to the disease. Progression-free survival was registered according to time interval from diagnosis to the inguinal region recurrence or distant metastasis. The actuarial survival and the overall survival for patient groups were calculated using the Kaplan-Meier method, while the difference in survival was assessed using the log-rank method. The impact of factors to overall survival was calculated with the Cox regression model in order to determine their independent contribution to the risk of death. Cox regression analysis was conducted in two steps. In the first step, univariate regression was estimated individually for each possible prognostic factor. In the second step, all prognostic factors from the univariate model were entered into a forward stepwise selection routine. Statistical significance in all cases was defined as *P* <0.05. The analysis was performed using the Statistical Packet for Social Sciences (SPSS, version 13.0). (SPSS Inc, Chicago, IL).

## Results

The five-year overall actuarial survival according to stage was 100% for clinical stage 1a and 81.5% for stage 1b. Of the 35 cases, the postoperative incision infection rate was 33.4%; severe complications such as rubber leg were not observed in the follow-up period. Five patients (14.3%) had tumor recurrence at 3.7 to 297.7 (median of 79.7) months after surgery. One patient died due to the recurrence of lung and mediastinal lymph node metastasis. One patient had a recurrence in the former location of the vulva near the urethra, and three patients had a limited recurrence in the vulva. After recurrence, a second surgical treatment was given. Surgical dissection of relapsed inguinofemoral lymph nodes or wide excision of vulvar recurrence was performed in case of local or regional recurrence. The patient with the recurrence near the urethra was treated with radiotherapy preoperatively; dosages that were delivered to the vulvar and inguinofemoral area ranged from 50–60 Gy.

All the results concerning the log-rank test for both overall and disease-free survival rates for the tested potential prognostic factors are shown in Table 1. Results of multivariate Cox regression analysis regarding the impact of factors to overall survival are given in Table 2. According to the univariate analysis, tumor location (lateral/medial), the general tumor type, clinical state of the inguinal lymph node and surgical procedure significantly influenced overall survival. These factors were subsequently tested on a multivariate model in terms of stepwise Cox-regression analysis. Thus, significant effect on overall survival and disease-free survival was due to surgical procedure (*P* = 0.005).

**Table 1:** Univariate analysis of overall survival and progression-free survival time

**Risk factors**	**Overall survival**			**Progression-free survival**	
	**Number of patients**	**OS at five years (%)**	**Log-rank*P*value**	**PFS at five years (%)**	**Log-rank*P*value**
**Age (years)**			**0.661**		**0.941**
≤50	6	83.3		83.3	
50 to 60	14	91.7		91.7	
>60	15	78.6		71.4	
**FIGO stage**			**0.733**		**0.637**
1a	5	100		100	
1b	30	81.5		77.8	
**Tumor location**			**0.047**		**0.039**
Lateral	9	64.8		66.7	
Medial	26	87.0		87	
**Grade**			**0.493**		**0.458**
I	26	84.0		84.0	
II	7	62.5		83.3	
III	2	50.0		50.0	
**Morphology**			**0.028**		**0.042**
cauliflower	26	91.3		87.0	
nodular	9	66.7		62.5	
**Lymph node clinical state**			**0.014**		**0.022**
not increased and active	16	73.3		66.7	
increased and hard	17	92.9		92.9	
syncretic	2	50.0		50.0	
**invasion depth (mm)**			**0.842**		**0.918**
≤1	5	100		100	
1 to 4	10	80.0		70.0	
5 to 7	10	62.5		62.5	
>7	10	75.0		75.0	
Surgical treatment			**0.005**		**0.003**
Local excision	2	83.9		83.9	
Radical vulvectomy and en bloc resection of inguinofemoral lymph nodes	12	81.8		72.7	
Radical vulvectomy and en bloc resection of inguinofemoral and pelvic lymph nodes	21	50.0		50.0	

FIGO, International Federation of Gynecology and Obstetrics; OS, overall survival; PFS, progression-free survival.

**Table 2:** Multivariate analysis of overall survival and progression-free survival time

**Prognosis**	**Factor**	**B**	**SE**	**Wald**	**df**	**Sig**	**Exp(B)**	**95% CI for Exp(B)**	
								**Lower**	**Upper**
OS	**Surgical treatment**	1.086	0.389	7.787	1	0.005	2.961	1.381	6.349
PFS	**Surgical treatment**	1.279	0.404	10.029	1	0.002	3.593	1.628	7.929

OS, overall survival; PFS, progression-free survival.

## Discussion

Primary cancer of the vulva is the most uncommon of all gynecological malignancies. It frequently occurs as inguinal lymph node metastasis and can invade urinary and reproductive tracts, thereby requiring, radical vulvectomy and en bloc resection of inguinofemoral lymphadenectomy. However, morbidity is impressive, seriously affecting a patient's quality of life. Early vulvar cancer confined to the vulva and with no lymph node metastasis needs much more individualized treatment. Recent research suggests that tumor stage, size, location, differentiation, depth of invasion and tumor-free margins are significant prognostic indicators [2,3]. However, because vulvar cancer is rare, randomized studies are also rare, and identification of prognostic factors remains controversial. Andreasson *et al*. concluded that age and tumor differentiation are among the clinical variables with significant prognostic value [4]. Moreover, Franckman *et al*. and Kosary reported a negative impact of age on survival [5]. Podratz *et al*. developed a model containing clinical stage, positive groin nodes and tumor size but not age as prognostic factors [6]. Boyce *et al*. included only the clinical stage [7]. A study by the Gynecologic Oncology Group (GOG) showed that the number of positive lymph nodes is the most important independent prognostic factor [8]. Our research involved only early vulvar squamous cell carcinoma (35 patients, all Stage 1 cases). The results showed the median survival time of the inguinal lymph node not increased and active were 162.6, and increased, active and hard were 164.0 and syncretic were 58.7 months. Five-year survival rates were 73.3%, 92.9% and 50% (*P* <0.05), respectively. Although clinical examination in judging the inguinal lymph node metastasis situation has certain limitations, the increased, active and hard lymph node status had better prognosis, suggesting that carcinoma cells caused a strong immune response in the lymph system, which can be more effective in killing tumor cells.

**Figure 1:**
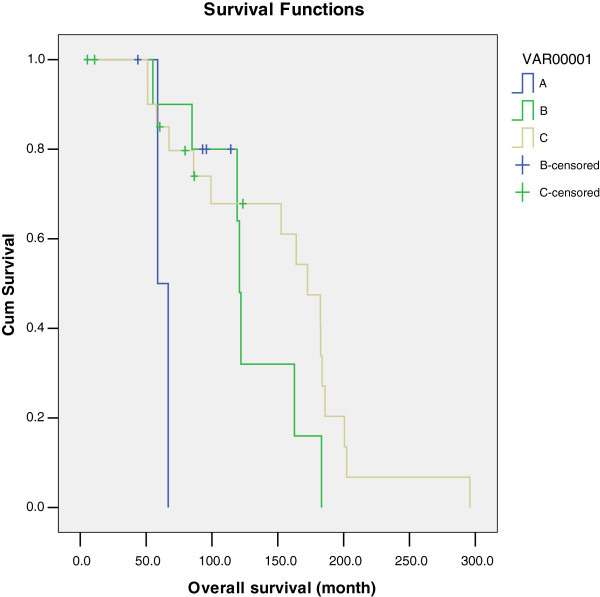
**Overall survival of early-stage squamous cell carcinoma of the vulva with different surgical treatment. A** stands for local excision. **B** stands for radical vulvectomy and en bloc resection of inguinofemoral lymph nodes. **C** stands for radical vulvectomy and en bloc resection of inguinofemoral and pelvic lymph nodes.

In the process of treatment, Taussig (1940) and Way (1948) presented a standard operation for cancer of the vulva (that is, vulvar radical resection and inguinal lymph node dissection), and the five-year survival rate for patients on whom this procedure is performed is approximately 70% [1]. Early stage vulvar squamous cell carcinoma confirmed no inguinal lymph node metastasis pathologically, but a 11% to 43% probability of lymph node micrometastasis exists [9]; Gonzalez and Bosquet reported a retrospective study of 320 patients with vulvar cancer who had undergone surgery; of all 214 cases without suspicious inguinal lymph node metastasis, 48 patients (22.4%) exhibited occult lymph node metastases [10]. In these cases it is necessary to resect the bilateral inguinal lymph node. Ali Ay Hand also concluded that the primary operation mode is the most important prognostic factor for patients, yet is also the only independent factor [11]. In this group of cases, in addition to two patients with small vulvar lesions that required local excision, the remaining patients had unilateral or bilateral inguinal lymph node dissection. The group requiring inguinal lymph node dissection comprised 1) nine cases of lateral-type tumors, 2) six cases of unilateral lymph node dissection, 3) two cases of small genital lesions that required local excision, 4) one case of preoperative examination and imaging suggestive of swollen contralateral lymph nodes and requiring bilateral lymph node dissection, and 5) 26 cases of medial type tumors that required bilateral lymph node dissection. Survival analysis revealed that surgical approach is an important factor affecting the prognosis and is also an independent prognostic factor for overall survival (*P* = 0.005) and progression-free survival time (*P* = 0.002), as shown in Figure 1. Our study shows the five-year survival of the patients who underwent wide local excision plus en bloc resection of inguinofemoral lymphadenectomy, or who underwent wide local excision plus en bloc resection of inguinofemoral lymphadenectomy and pelvic lymph nodes was 81.8% and 83.9% (*P* >0.05). Therefore pelvic lymph node dissection does little to help patient survival and is no longer considered necessary surgery in most cases. Since FIGO began the new system of stage identification in 2009, the size of the lesion no longer serves as the basis of inguinal lymph node excision for early vulvar cancer; depth of invasion is a better determinant of the potential for lymph node metastasis [12].

**Figure 2:**
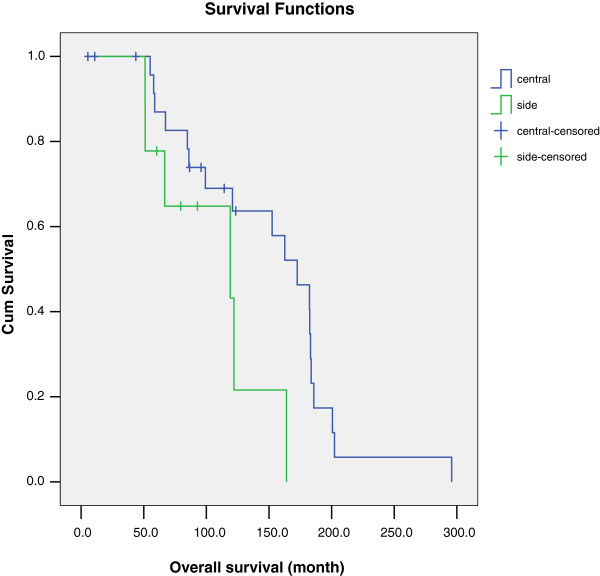
Overall survival of early-stage squamous cell carcinoma of the vulva with different location.

**Figure 3:**
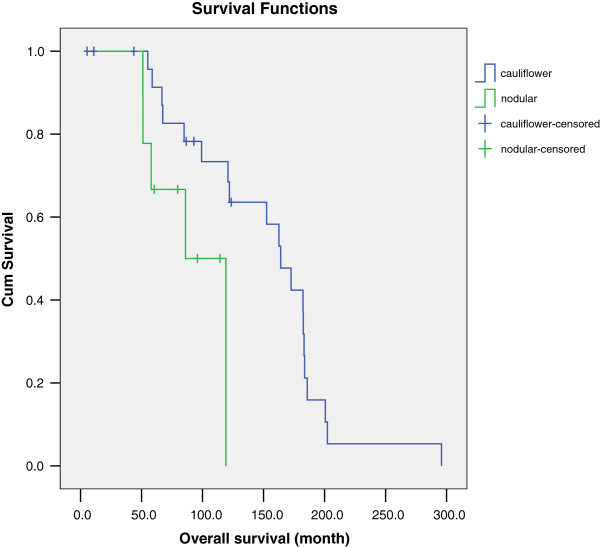
Overall survival of early-stage squamous cell carcinoma of the vulva with different tumor type.

According to our results, the location of the tumor is one of the important factors in early vulvar squamous cell carcinoma. The median survival time in medial type and lateral type of tumor were 172.5 months and 119.2 months; five-year survival rates according to tumor location were 87.0% and 64.8% (*P* <0.05) as shown in Figure 2. Research has reported that medial tumors develop faster and are more prone to transfer so the prognosis is relatively poor. But most of this research concerns all stages of vulvar tumors, whereas our data involve only the early vulvar squamous cell carcinoma. It is possible that early cancer without lymph node metastasis progresses slowly but it is also possible that since medial types are located close to the urethra and vagina, thereby easily causing symptoms for patients, they can be identified earlier and with a better prognosis than the lateral type. This might be worth exploring in future research involving more cases. In our study, we also found that the gross preoperative morphology type is cauliflower-like, a type which has a better prognosis than the nodular type. The median survival time by morphology type was 164 months and 86 months with five-year survival rates of 91.3% and 66.7% (*P* <0.05), respectively (Figure 3). Nodular tumors are generally endogenous growth, exhibit late onset of symptoms, and easily infiltrate the lymphatic vessels, whereas cauliflower tumors tend to grow outside and invade the lymph tube later so the prognosis is better. Research on morphology types are still relatively lacking, however, and further randomized studies are needed to confirm these assumptions.

In early stage vulvar carcinoma patients, there is at trend towards individualized treatment in order to reduce morbidity and sequelae following inguinal lymph node dissection. However, recurrences in the groin are almost always fatal. A variety of modifications in groin treatment have been introduced [13]. For patients with a stage 1a, tumor confined to the vulva with a maximum diameter of ≤2 cm and an infiltration depth ≤1 mm, there is no need to excise the inguinal lymph node [14]. Because the risk of lymph node metastasis is negligible (<1%), a wide local excision can be used. For stage 1b of a lateral type case, the occurrence of contralateral inguinal lymph node metastasis risk is relatively low, and may therefore require only resection with ipsilateral lymph node without the contralateral lymph node. Stage 1b medial type or large unilateral lesions (diameter >2 cm) still require bilateral inguinal lymph node excision. Homesley [15] reported that if tumor invasive depth was ≤2 mm, ipsilateral inguinal lymph node positive rate was 61.8%, whereas for contralateral lymph nodes without metastasis but with an infiltration depth of 3 to 5 mm, contralateral lymph node positivity rate was 11.9%, and if infiltration depth was 6 to 10 mm, contralateral lymph node positivity rate was 31.8%. Therefore the author thinks that, on the premise of individual treatment, en bloc resection of inguinofemoral lymphadenectomy can reduce recurrence and metastasis rate.

For early cases, the availability of pelvic and inguinal area radiotherapy instead of en bloc resection of inguinofemoral lymphadenectomy has become a focus of debate. Perez concluded that early stage vulvar carcinoma treated by radiotherapy alone or with local excision plus adjuvant postoperative radiotherapy, can effectively reduce the incidence of complications, whereas efficacy with traditional surgery showed no difference [16]. A prospective study by GOG randomly divided patients with negative lymph nodes into a simple inguinal radiotherapy group and an inguinal lymph node resection group. Five out of 27 patients (18.5%) who received primary groin radiation developed a groin recurrence, which is an unacceptably high failure rate, and therefore the trial was closed in advance of its original accrual goal [17]. In 2002, Van der Velden and Ansink published a review in which they concluded that primary radiotherapy to the groin results in less morbidity but also results in a higher number of groin recurrences compared with surgery. They suggested that an operation was the preferred treatment option for early vulvar cancer patients [18]. But a large sample, single institution experience by Katz showed the groin recurrence rate after primary radiotherapy was similar to only superficial inguinal lymphadenectomybut in a higher groin recurrence rate compared with complete inguinal lymphadenectomy [19]. As a result, a large sample, prospective study is needed to confirm whether or not inguinal radiation is safe for early cancer treatment.

## Competing interests

The authors declare that they have no competing interests.

## Authors’ contributions

LQX designed this scientific research, analysed data and drafted the manuscript,While RZL and HJH provided pathological technical guidance,and XMS offered the author with the basic knowledge of radiation, YNZ is the mentor. All authors read and approved the final manuscript.
